# Neuraxial anesthesia for patients with severe pulmonary arterial hypertension undergoing urgent open abdominal surgeries: two case reports

**DOI:** 10.1186/s40981-024-00737-w

**Published:** 2024-09-02

**Authors:** Shuhei Yamada, Yoshiaki Takise, Yuri Sekiya, Yuya Masuda, Yoshi Misonoo, Kenta Wakaizumi, Tomohiro Suhara, Hiroshi Morisaki, Jungo Kato, Takashige Yamada

**Affiliations:** Department of Anesthesiology, Keio University School of Medicine, Tokyo, 1608582 Japan

**Keywords:** Pulmonary hypertension, Adult congenital heart disease, Noncardiac surgery, Neuraxial anesthesia

## Abstract

**Background:**

There is no consensus regarding the choice of anesthetic method for patients with pulmonary hypertension (PH). We report two cases in which neuraxial anesthesia was safely performed without general anesthesia during open abdominal surgery in patients with severe PH.

**Case presentation:**

Case 1: A 59-year-old woman had an atrial septal defect and a huge abdominal tumor with a mean pulmonary arterial pressure (PAP) of 39 mmHg and pulmonary vascular resistance (PVR) of 3.5 Wood units. Case 2: A 23-year-old woman who had hereditary pulmonary artery hypertension (mean PAP, 65 mmHg; PVR, 16.45 Wood units). Both patients underwent open abdominal surgery under neuraxial anesthesia without circulatory collapse with intraoperative administration of vasoconstrictors.

**Conclusion:**

Although anesthetic care must be personalized depending on the pathology and severity of PH, neuraxial anesthesia may be an option for patients with severe PH undergoing abdominal surgery.

## Background

Pulmonary hypertension (PH), which is defined as an elevated mean pulmonary arterial pressure (PAP) > 20 mmHg at rest [1], poses great challenges in anesthetic management. Patients with severe PH are reported to be at higher risk of perioperative morbidities, such as congestive heart failure, hemodynamic instability, sepsis, and right heart failure [2]. These morbidities lead to the high perioperative mortality in the range of 4 to 24% in patients with severe PH [3]. Currently, there is no consensus on the choice of anesthetic method for patients with severe PH. Here, we present two patients with severe PH with different pathologies who underwent urgent open abdominal surgery. In both patients, successful circulatory management was achieved under neuraxial anesthesia.

## Case presentation

### Case 1

**Fig. 1:**
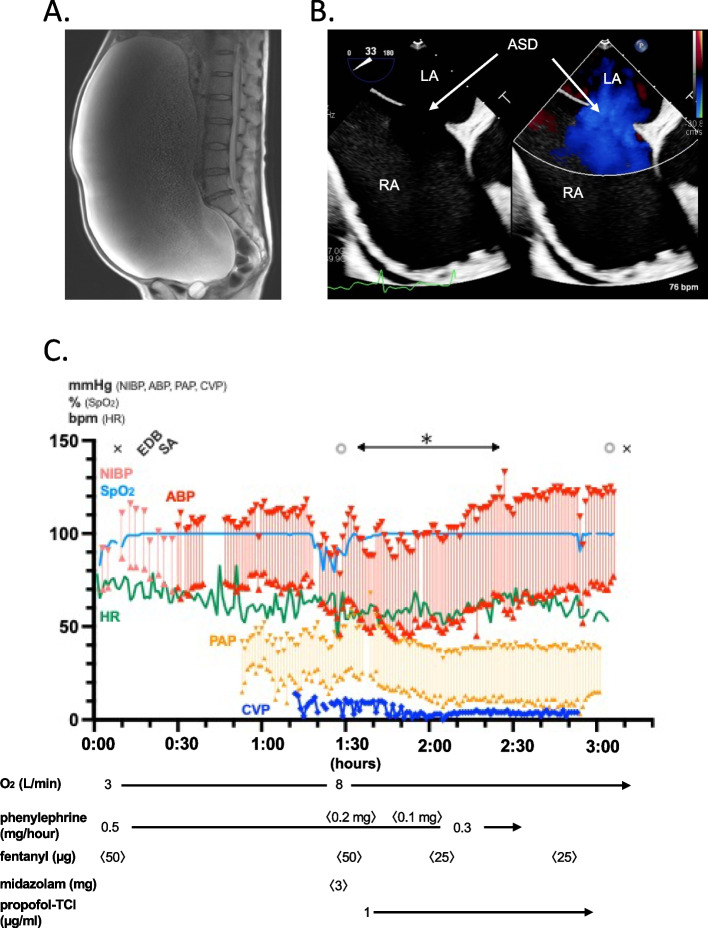
Preoperative images and intraoperative anesthetic course in Case 1. **A** Sagittal view of T2-weighted MRI showing a large ovarian tumor (38 × 20 × 37 cm). **B** Atrial septal view of transthoracic echocardiography. Atrial septal deficit with 26-mm diameter and left–right shunt were identified. RA, right atrium; LA, left atrium; ASD, atrial septal deficit. **C** Hemodynamic parameters during open abdominal surgery under neuraxial anesthesia. After epidural and subarachnoid anesthesia, surgery started while monitoring ABP, CVP, and PAP. Contents in the tumor were aspirated in 500-mL increments, and a total of 15 L of serous fluid was aspirated over 30 min within the period (*). CVP and PAP decreased, whereas ABP and pulse pressure increased with the aspiration. × , anesthesia start/end; EDB, epidural block; SA, spinal anesthesia; ◎, surgery start/end

A 59-year-old woman (height, 163 cm; weight, 65 kg) with an untreated atrial septal defect (ASD) was hospitalized with complaints of abdominal distention, leg edema, and dyspnea. Computed tomography revealed a giant ovarian tumor occupying the entire abdominal cavity (Fig. 1A). An urgent gynecological surgery was scheduled. Laboratory data showed an elevated brain natriuretic peptide (BNP) of 621 pg/mL, and a preoperative right heart catheter examination revealed severe PH, presumably associated with ASD, with a mean PAP of 39 mmHg, a high pulmonary blood flow to systemic blood flow ratio (Qp/Qs) of 2.7, and high pulmonary vascular resistance (PVR) 3.5 Wood units. Transesophageal echocardiography showed a large ASD (diameter, 26 mm; secondary type; Fig. 1B) with a left-to-right shunt, dilated right atrium and ventricle, moderate pulmonary regurgitation, and moderate tricuspid regurgitation (TR). Given the limited free space in the abdominal cavity owing to the large tumor size, open surgery was chosen over the laparoscopic approach. Considering the potential hazardous effects of general anesthesia and mechanical ventilation on pulmonary and systemic circulation in case of severe PH and the risk of regurgitation of gastric contents during intubation, we decided to avoid general anesthesia and performed combined spinal-epidural anesthesia (CSEA).

Upon arrival in the operating room, standard vital monitors, including an electrocardiogram, a pulse oximeter, and a noninvasive blood pressure monitor, were placed. The peripheral oxygen saturation (SpO2) before the administration of oxygen was 93 to 97%. To counteract the hypotension caused by the neuraxial block, an intravenous infusion of phenylephrine (1 mg/h) was initiated before starting CSEA. An epidural catheter was placed in the T11–12 interspace. Consequently, spinal anesthesia was induced at the L4–5 interspace, and 2.5 mL of 0.5% isobaric bupivacaine was administered intrathecally. Successful sensory blockade above the T4 level was confirmed through a cold test with an alcohol swab. Furthermore, an arterial catheter was inserted into the right radial artery, and a pulmonary artery catheter was inserted under local anesthesia to monitor arterial blood pressure (ABP), PAP, and central venous pressure (CVP), respectively. Surgery was initiated after achieving conscious sedation with boluses of midazolam and fentanyl and a target-controlled infusion of propofol. Although additional boluses of phenylephrine were required to maintain the ABP, however, the CVP and PAP remained stable. In total, 15 L of intra-tumor fluid was carefully aspirated over 30 min. Meanwhile, the ABP gradually increased, whereas the mean PAP and CVP decreased. The phenylephrine infusion was discontinued after aspiration (Fig. 1C). The tumor was completely resected, and the operation was completed within 90 min. The intraoperative fluid infusion volume, blood loss, and urine output were 823, 50, and 300 mL, respectively. The postoperative course was uneventful with no remarkable postoperative pain or hemodynamic derangement. The patient was discharged from the hospital on postoperative day (POD) 9.

### Case 2

**Fig. 2:**
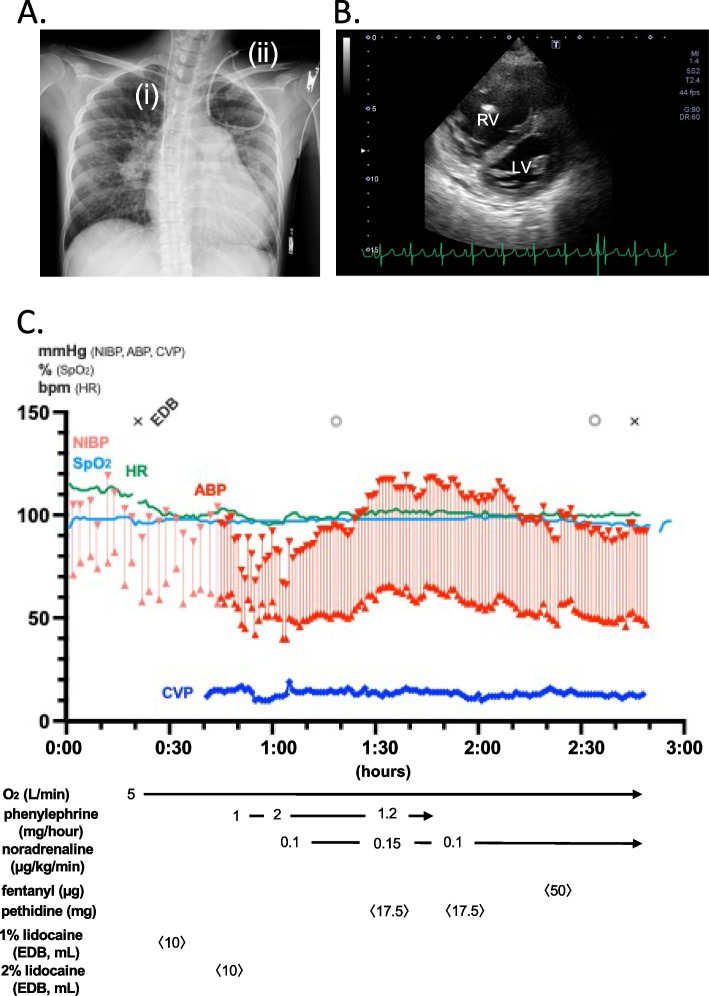
Preoperative assessment and intraoperative anesthetic course in Case 2. **A** Preoperative chest radiography after insertion of the central venous catheter. Central venous catheter (i) was inserted from the right internal jugular vein for perioperative catecholamine infusion, in addition to Hickman catheter (ii) inserted from the left internal jugular vein into the right atrium for a continuous infusion of a vasodilator. **B** The transgastric mid-papillary short-axis view of the preoperative transesophageal echocardiography. Right ventricular enlargement and compression of the left ventricle were identified. RV, right ventricle; LV, left ventricle. **C** Hemodynamic parameters during open abdominal surgery under neuraxial anesthesia: During surgery, pethidine and fentanyl were administered intravenously for stimuli of peritoneal traction. × , anesthesia start/end; EDB, epidural block; ◎, surgery start/end

A 25-year-old woman (height, 151 cm; weight, 42 kg) with hereditary pulmonary arterial hypertension was admitted to our hospital with upper abdominal pain. An acute cholecystitis was diagnosed, and the surgical team planned an emergency cholecystectomy. The right heart catheter examination performed 5 months earlier showed a high PAP of 92/48 (mean, 65) mmHg, a significantly elevated PVR of 16.45 Wood units, and a cardiac index of 2.65 L/min/m^2^. Despite home oxygen therapy (2 L/min) and continuous infusion of treprostinil at 220 ng/kg/min into the right atrium (Fig. 2A), she had been suffering from dyspnea on effort. Transthoracic echocardiography performed after hospitalization revealed a markedly enlarged right ventricle compressing left ventricle (Fig. 2B). Considering the limited reserve of the pulmonary circulation, the multidisciplinary team decided to perform open abdominal surgery under epidural anesthesia, avoiding general anesthesia.

In the operating room, the baseline SpO2 with 2 L/min of oxygen via nasal cannula was 95 to 97%. An epidural catheter was inserted from T6 to 7, and 10 mL of 1% lidocaine was administered. In addition, an arterial catheter through the right radial artery and a central venous catheter through the right internal jugular vein were secured under local anesthesia. After confirming sensory loss above the T4 level, surgery was initiated (Fig. 2C). Intraoperatively, as the patient occasionally complained of discomfort, bolus infusions of 50 µg of fentanyl and 35 mg of pethidine in total, and an additional epidural injection of 10 mL of 2% lidocaine, were required. The intraoperative ABP was generally maintained by continuous administrations of phenylephrine (1–2 mg/h) and noradrenaline (0.1–0.15 µg/kg/min). The surgery was completed in 88 min. The intraoperative fluid infusion volume, blood loss, and urine output were 646, 90, and 150 mL, respectively. Postoperative pain was well-controlled with patient-controlled epidural analgesia. Her hemodynamic status remained stable postoperatively, and noradrenaline administration was discontinued on POD1. The patient recovered uneventfully and was discharged from the hospital on POD14.

## Discussion

Severe PH complicates anesthetic management by potentially triggering a vicious cycle as follows: (1) worsening of right heart failure, (2) reduced forward flow to the left heart, and (3) decreased cardiac output and reduced coronary perfusion, further deteriorating right heart failure [4]. Therefore, the major goals of perioperative hemodynamic management for patients with severe PH include avoiding further elevation of PVR and maintaining cardiac contractility and systemic perfusion pressure.

Concerning abdominal surgeries, the choice of surgical approach may have a great impact on hemodynamics in patients with severe PH. In both of our cases, multidisciplinary discussions eventually led to the selection of the open approach over the laparoscopic approach. Despite previous reports demonstrating successful laparoscopic surgeries for patients with severe PH [5–7], the implementation of pneumoperitoneum in the laparoscopic approach potentially worsens PH through several mechanisms, such as abdominal distention, hypercapnia, acidemia, and reduced lung compliance [8, 9]. Although the invasiveness and postoperative pain associated with the open approach can be deleterious, these disadvantages were successfully overcome by postoperative epidural analgesia in both of our cases. Thus, an open approach can be an option for patients with severe PH provided that excellent postoperative pain control can be achieved with epidural analgesia.

Several predisposing factors have been proposed for predicting poor postoperative outcomes after noncardiac surgeries in patients with severe PH. These include age $\ge$ 75 years, New York Heart Association functional classification $\ge$ II, systolic PAP $\ge$ 70 mmHg, CVP > 7 mmHg, emergency cases, and moderate-to-high-risk surgical procedures [10–13]. In the case of adult congenital heart disease with PH, various postoperative risk stratification models for noncardiac surgeries based on multiple factors, such as physical status, complexity of the cardiac lesion, age, other comorbidities, and risk of the surgical procedure, have also been validated [14]. Recently, Hassan et al. proposed a simple composite risk assessment tool for patients with PH undergoing noncardiac surgery that combines patient-level and procedural variables (Table 1) [15]. Based on these risk stratifications, our cases were both classified into the high-risk group.

**Table 1:** A simple perioperative risk assessment tool for patients with pulmonary hypertension [15]

Variables	Score
WHO functional class	
I or II	0
III or IV	1
6-min walking distance	
> 400 m	0
≦400 m	1
BNP or NT-proBNP	
BNP < 50 ng/L or NT-proBNP < 300 ng/L	0
BNP ≧ 50 ng/L or NT-proBNP ≧ 300 ng/L	1
AHA/ACC procedural risk	
Low	0
Elevated	4

0–2, low risk; 3, intermediate risk; 4–7, high risk

Nevertheless, the pathology of PH can change dynamically in response to anesthetic and surgical interventions. As for adult congenital heart diseases with intracardiac shunts, such as in Case 1, a worsening of PH may increase the right-to-left shunt, immediately leading to systemic deoxygenation. Therefore, regardless of the preoperative risk estimation, close perioperative monitoring, including ABP, CVP, pulse oximetry (SpO2), PAP, and echocardiography, should always be considered in patients with severe PH. Although the aspiration of 15 L of intra-tumor fluid over 30 min did not result in major hemodynamic changes in Case 1, such a radical surgical maneuver should have been carefully performed with meticulous monitoring of the aforementioned hemodynamic parameters.

The choice of anesthetic method remains a topic of discussion for patients with severe PH. Although general anesthesia can provide excellent depth of sedation, muscle relaxation, and analgesia, the most commonly used general anesthetics have hemodynamically unfavorable effects on PH circulation, such as reductions in cardiac contractility, systemic vascular resistance (SVR), and preload [6, 16]. In addition, an increase in airway pressure caused by mechanical ventilation potentially aggravates PH by elevating the PVR [17]. Moreover, sympathetic responses associated with intubation and extubation may have detrimental effects on the PH circulation. However, some guidelines recommend not using spinal anesthesia in patients with severe PH because of concerns regarding rapid hemodynamic derangements, including the rapid reduction of SVR and preload [13, 18]. Despite these recommendations, the prophylactic use of phenylephrine infusion successfully counteracted spinal anesthesia-induced hypotension in Case 1. Hemodynamically, epidural anesthesia shares similar disadvantages with spinal anesthesia but generally has a milder and slower onset of sympathetic inactivation. However, in Case 2, a higher dose of phenylephrine and noradrenaline infusion were required to maintain the ABP. The severer PH in Case 2 (mPAP, 39 mmHg, PVR 3.5 Wood units in Case 1; mPAP, 65 mmHg, PVR 16.5 Wood units in Case 2) may have contributed to the more pronounced hemodynamic effects of neuraxial anesthesia.

Vasoactive drugs are often necessary to counter the hypotension associated with anesthesia in patients with severe PH. However, the selection of vasoactive drugs should be determined with special caution. In our case, maintenance of ABP was achieved with vasopressors, including phenylephrine and noradrenaline, without any signs of hemodynamic derangement. However, these vasopressors potentially disrupt circulation in PH by increasing PVR via the action of alpha-adrenergic receptors in the pulmonary vasculature [13]. Although there are few data, vasopressin can be an option in some cases of severe PH because of its systemic vasopressive effect without increasing PVR [3, 11]. The use of inotropes, including dobutamine, PDE-III inhibitors, adrenaline, and/or inhaled nitric oxide, should be considered in patients with progressive right ventricle dysfunction.

Collectively, although the use of vasopressors should be optimized depending on the severity, pathophysiology, and responsiveness to PH, neuraxial anesthesia may be an option for patients with severe PH undergoing abdominal surgery. Nevertheless, rigorous hemodynamic monitoring and multidisciplinary collaboration are mandatory for the safe perioperative management of patients with severe PH.

## Data Availability

The data in this case report are available from the corresponding author on reasonable requests.

## Abbreviations

PH: Pulmonary hypertension
PAP: Pulmonary arterial pressure
ASD: Atrial septal defect
BNP: Brain natriuretic peptide
RHC: Right heart catheter
Qp/Qs: Pulmonary blood flow to systemic blood flow ratio
PVR: Pulmonary vascular resistance
TR: Tricuspid regurgitation
CSEA: Combined spinal-epidural anesthesia
SpO2: Peripheral oxygen saturation
ABP: Arterial blood pressure
CVP: Central venous pressure
POD: Postoperative day
SpO2: Pulse oximetry
SVR: Systemic vascular resistance
RA: Right atrium
LA: Left atrium
EDB: Epidural block
SA: Spinal anesthesia
RV: Right ventricle
LV: Left ventricle
